# Surgical treatment of syphilitic superficial femoral artery aneurysm: a rare case report

**DOI:** 10.1186/s12879-015-1239-7

**Published:** 2015-11-20

**Authors:** Zhihua Cheng, Sean X. Luo, Xiwei Sun, Siqiao Sun, Zhongying Wang, Yuzhu Jiang, Zhiyuan An, Qi Wang

**Affiliations:** Department of Vascular Surgery, the First Hospital of Jilin University, 71 Xinmin Street, Changchun, 130021 Jilin Province China; Department of Vascular Surgery, Panshi City Hospital, Panshi, 132300 Jilin Province China

**Keywords:** Syphilis, Femoral artery aneurysm, Stent implantation, Surgery resection

## Abstract

**Background:**

Arterial aneurysm is a known complication of syphilis, but the occurrence of femoral artery aneurysm secondary to the syphilitic disease has never been reported.

**Case presentation:**

The present study described a 60-year-old Chinese male who presented with two aneurysms in the middle and lower segment of the right superficial femoral artery causing the symptoms of pain, coldness and numbness in the right lower limb. This case was diagnosed with syphilitic superficial femoral aneurysm because of positive syphilitic testing and the inflammatory cell infiltration around the adventitial vasa vasorum under the pathological examination. Anti-syphilis treatment, stent graft implantation and open surgery were attempted to eliminate the syphilis and aneurysm, which was ultimately successful, with no symptoms after a follow-up of 3 months.

**Conclusion:**

Combined open and endovascular repair may be effective and safe for treatment of syphilitic femoral artery aneurysms.

## Background

Syphilis caused by the spirochete bacteria *Treponema pallidum* subspecies *pallidum* is one of the most common sexually transmitted diseases in China [1, 2]. It is reported that China has recently been experiencing a mounting epidemic of syphilis infection, showing a rise in incidence from 0.2 per 100,000 in 1993 to 32.04 per 100,000 in 2011 [3]. Tertiary (late) syphilis is known to cause an inflammatory proliferation, invasion of the adventitia vasa vasorum, subsequent fibrosis and calcification of the arterial wall, leading to the possible formation of aneurysms. The syphilitic aneurysms can occur in almost any artery, but are most commonly found in the aorta, with the ascending aorta as the most frequently involved segment (in 50 % of cases), followed by the aortic arch (in 35 % of cases), the descending aorta (in 15 % of cases) and the abdominal aorta (uncommon). Syphilitic aneurysm is scarcely seen in peripheral arteries [4, 5], with no report about the occurrence of femoral artery aneurysm secondary to the syphilitic disease. Here, we described a rare case of syphilitic femoral aneurysm and discussed the treatment procedure for this patient.

## Case presentation

**Fig. 1:**
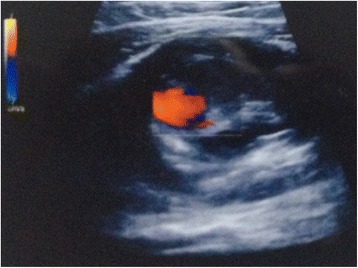
The color Doppler ultrasonography of the right lower limb showed the presence of the aneurysm at the right femoral superficial artery accompanied by peri-hematoma

**Fig. 2:**
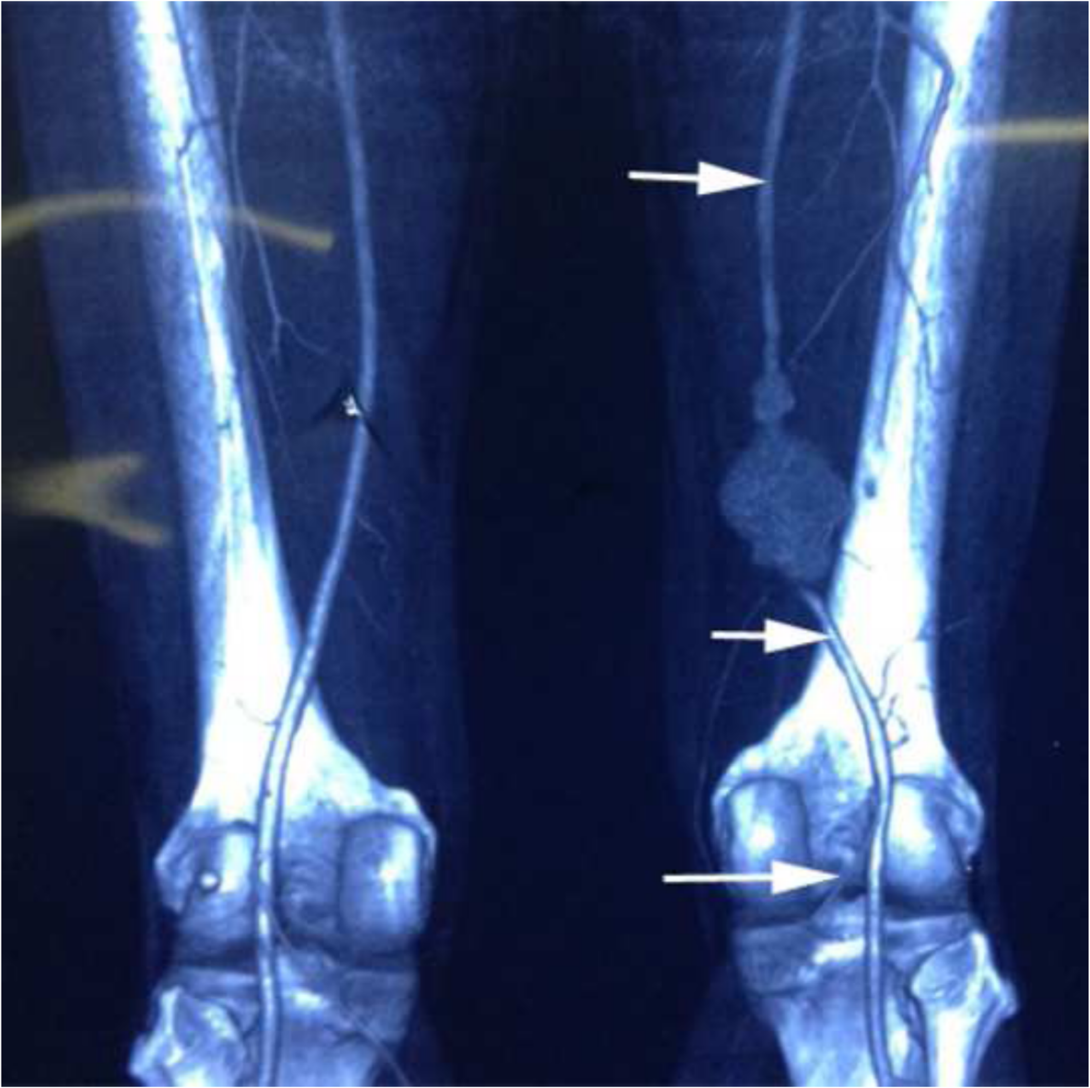
The computed tomography angiography of the lower limbs showed the formation of two aneurysms in the middle and lower segment of the right femoral superficial artery (arrow)

A 60-year-old Chinese male presented to our hospital complaining of pain, coldness and numbness in the right lower limb for one month. On physical examination, he was afebrile and had a pulsatile, firm subcutaneous mass with a diastolic and systolic murmur on the medial aspect of the right thigh. Peripheral pulsations could be palpated well at the right common femoral artery, but not the popliteal artery, dorsalis pedis artery and the tibialis posterior artery, indicating the occlusion of superficial femoral artery (SFA). Additional imaging, including the color Doppler ultrasonography (CDUS, Fig. 1) and computed tomography angiography (CTA, Fig. 2) conducted to evaluate the potential embolic sources, revealed the formation of two aneurysms in the middle and lower segment of the right SFA [6], accompanied by the presence of mural thrombus. The proximal aneurysm was located higher than the Hunter’s canal with the maximal diameter of 15 mm, while the distal aneurysm (which was ruptured, but only with localized hematoma, not progressive bleeding) was located within the Hunter’s canal with the maximal diameter of 26 mm. The normal neurological, cardiovascular systems examinations and no significant medical history of hypertension, heart diseases, diabetes mellitus, blood transfusion, genital ulcer and skin rash seemed to indicate our case was less possibly caused by the complications of these diseases. The popliteal lymph nodes were not palpated and clearly swollen in CDUS and CT examinations, excluding the lymph nodes origin. However, he recounted he had heterosexual extramarital unprotected sexual contacts twenty years ago, but denied any sexual activity since his wife died eighteen years ago. His wife had one previous abortion before death, but no syphilis testing was performed. These promoted us to perform the syphilis tests for this patient. As a result, the patient tested positive for *Treponema pallidum* hemagglutination antibody and rapid plasma regain (RPR, titer 1/16). The patient denied any recent inoculations, vaccinations or complementary treatments that may cause a false positive syphilis serology [7]. Additional laboratory tests revealed the elevated levels of both the erythrocyte sedimentation rate (ESR, 58 mm/1 h) and the serum C-reactive protein (CRP, 15.4 mg/L), suggesting the inflammatory arteritis. A presumptive diagnosis of right femoral aneurysm secondary to the tertiary syphilis from unprotected sexual contacts was given.

**Fig. 3:**
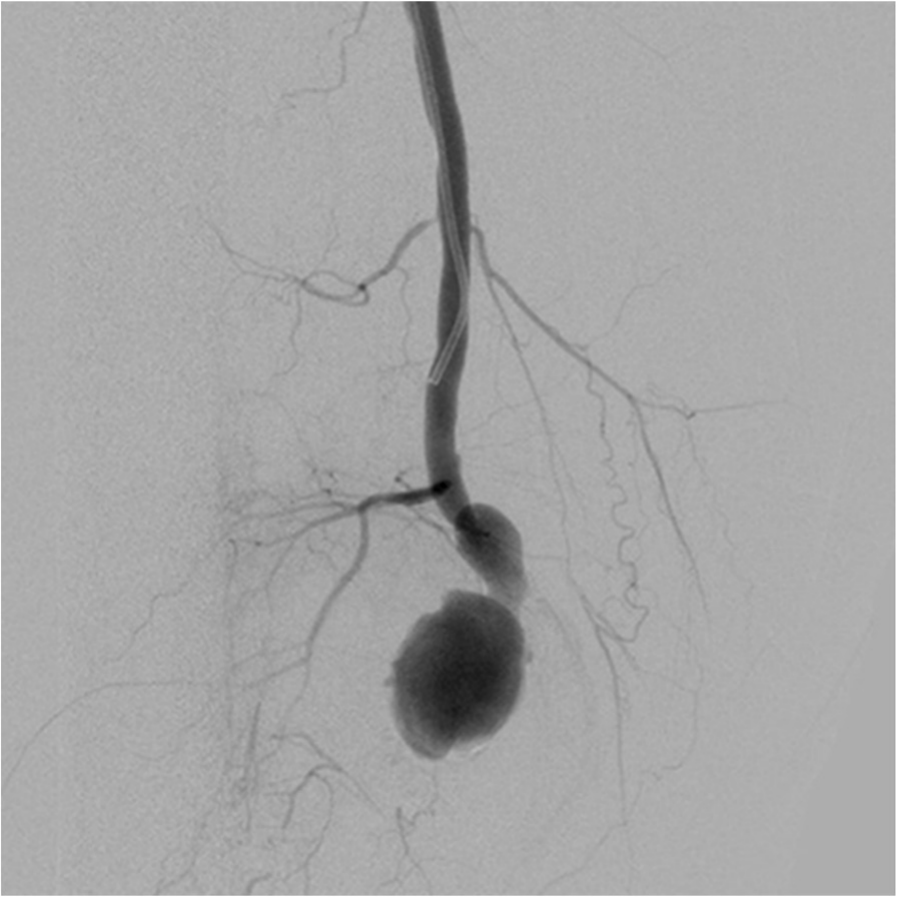
The digital subtraction angiography demonstrated the distal occlusion of the right femoral superficial artery

**Fig. 4:**
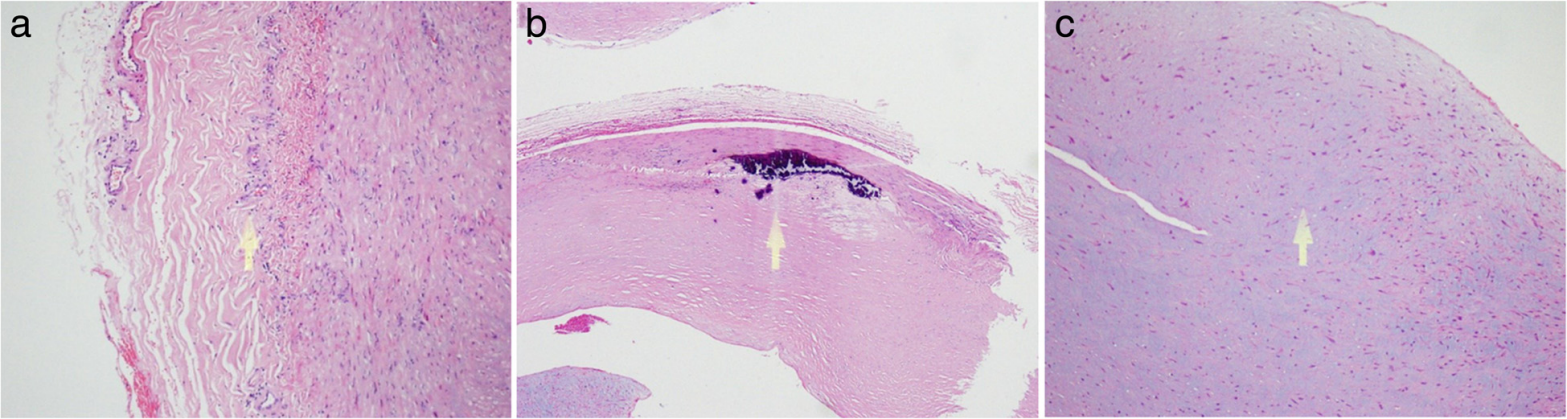
Pathology of the excised aneurysmal tissue revealed the proliferation of the vasa vasorum in the adventitia, with sporadic infiltration of inflammatory cells (arrow, **a**); the fibroplasia in the arterial wall along with the local calcification (arrow, **b**) and mucoid degeneration (arrow, **c**)

In view of the active syphilis infection and the high vascular inflammatory reaction that may cause pseudoaneurysm and rupture of the artery anastomosis if surgery was performed, the patient was firstly treated with benzathine penicillin (2.4 MU, i.m) once a week. Four weeks later, the titer of RPR, the concentration of CRP and ESR were respectively reduced to 1:12, 12.5 mg/L and 42 mm/1 h, demonstrating the effectiveness of anti-syphilis therapy. However, the patient presented aggravated pain at the right thigh, which was considered to result from the hematoma enlargement due to the rupture and hemorrhage of the aneurysm. Thus, the operation treatment was prepared. In an effort to minimize the morbidity and mortality of femoral aneurysm repair, implantation of the Viabahn covered stent graft (Gore & Associates) was performed to isolate the aneurysm [8]. Intraoperatively, a 90-cm, 6-F Flexor sheath (Cook) was advanced into the right common femoral artery in a retrograde fashion. Nevertheless, the distal occlusion of the right femoral artery was still displayed on the digital subtraction angiography (DSA, Fig. 3), which may be attributed to the tortuous arterial wall compressed by the aneurysm and hematoma. Then a single 5-F, cobra-shaped catheter (Terumo) combined with a 0.035-inch smooth Radiofocus hydrophilic guidewire (Terumo) were inserted but they could also not pass through the distal end of the aneurysm. Subsequently, an incision was made below the right medial malleolus to expose the right tibialis posterior artery and the retrograde puncture was performed to place a 5-F dilator catheter (Cook). A 0.018-inch guide wire (Boston Scientific) was inserted through the dilator, however, the distal end of the aneurysm was still not opened. The routine open operation via a medial incision was eventually carried out to remove the right femoral aneurysm under the general anesthesia. Pathological examination of the resected aneurysm showed the proliferation of adventitial vasa vasorum, with sporadic infiltration of inflammatory cells (Fig. 4a); the fibroplasia in the arterial wall accompanied by local calcification (Fig. 4b) and mucoid degeneration (Fig. 4c). Accordingly, the patient was ultimately confirmed with right femoral aneurysm due to syphilis.

Postoperatively, the anti-syphilis treatment continued. After three months, the laboratory findings indicated that RPR and *Treponema pallidum* hemagglutination antibody were negative. The CDUS demonstrated that the stenosis, occlusion and re-formation of aneurysm were all not found in the arteries of the right lower limb. The patient was also well and asymptomatic.

## Conclusions

Femoral artery aneurysms are rarely encountered peripheral aneurysms in clinic, with an incidence of approximately 5 per 100,000 population [9]. The occurrence of true aneurysms of the femoral artery is commonly associated with atherosclerosis [10], mycotic infection [11, 12] and autoimmune connective tissue disorders (i.e. Behcet’s syndrome) [13]. However, these etiologies were less likely based on the following observations: 1) this patient did not have the history of hypertension, diabetes mellitus and hyperlipidemia, which are the highly confirmed risk factors for arteriosclerosis [14]; 2) the CTA and CDUS results showed no signs of serious arteriosclerosis; 3) the aneurysm caused by arteriosclerosis is frequently solitary according to our limited clinical experience. However, the patient in our study had two aneurysms at the right superficial femoral artery; 4) no history and signs of genital ulcer and skin rash ruled out the Behçet’s disease [13]; 5) afebrile, unremarkable cardiac examination and negative culture results for bacteria and mycetes demonstrated the impossibility of endocarditis [15]. Subsequently, the patient acknowledged that he had the history of extra-marital unprotected sexual intercourses, which promoted us to suspect the syphilis mechanism. As expected, the positive serologic testing for *Treponema pallidum* hemagglutination antibody and RPR (titer 1/16) were found. After surgery, the pathological analysis showed the inflammatory cell infiltrates around the vaso vasorum of the adventitia, the fibrosis and calcification in the arterial wall (Fig. 4). These above findings conformed to the pathological changes of the syphilitic arteritis [16, 17]. Therefore, we believe the syphilitic arteritis contributes to the formation of femoral artery aneurysm.

It is reported that symptomatic syphilitic aneurysms may tend to progress rapidly and induce death from aneurysm rupture [18]. Thus, an urgent treatment of the aneurysm should be undertaken. Management of syphilitic femoral aneurysm involves resection of the affected artery, endovascular stenting together with long-term anti-syphilis treatment. Compared with the open surgery, the endovascular technique can avoid major open incisions, prolonged hospital stay, associated morbidity from limb dissection and scarring [19, 20]. In addition, the high risk of pseudoaneurysm and rupture of the artery anastomosis was present when open surgery was performed for our patient who manifested with high vascular inflammatory reaction. Consequently, the minimally invasive operation of implanting a coated stent graft was given our priority. Nevertheless, the operation was unsuccessful due to occlusion of the distal end of aneurysm. Therefore, conversion to open surgery was required to remove the distal femoral artery aneurysm under the general anesthesia. The excellent outcomes, including negative laboratory testing and no evidence of stenosis, occlusion, re-formation of aneurysm and clinical symptoms, confirmed the effectiveness of our treatment.

In conclusion, this may be the first report of a patient with syphilitic femoral artery aneurysm who was successfully treated by combined open and endovascular repair. However, this study has some limitations, such as the shorter follow-up time, the treponeme not identified in the removed tissues, and the syphilis confirmed only by RPR and *Treponema pallidum* hemagglutination antibody assay, not including other tests, such as venereal disease research laboratory (VDRL), treponemal pallidum particle agglutination (TPPA), fluorescence test assay absorption (FTA-ABS), the enzyme immunoassay (EIA) or the chemiluminescent immunoassay (CLIA) [21]. Thus, case series studies are needed to provide the systematic diagnosis and treatment recommendation for syphilitic femoral artery aneurysm.

## Consent

Written informed consent was obtained from the patient for publication of this Case report and any accompanying images.

## Abbreviations

CDUS: Color Doppler ultrasonography
CRP: C-reactive protein
CTA: Computed tomography angiography
DSA: Digital subtraction angiography
ESR: Erythrocyte sedimentation rate
PRR: Rapid plasma regain
SFA: Superficial femoral artery
